# Supplementary material for: The Transcription Factor CsgD Contributes to Engineered Escherichia coli Resistance by Regulating Biofilm Formation and Stress Responses
Source: Int J Mol Sci. 2023 Sep 5;24(18):13681. doi: 10.3390/ijms241813681 (PMC10530992; doi:10.3390/ijms241813681)
Supplement: Supplementary file 1 [file ijms-24-13681-s001.zip › ijms-2561868-supplementary.pdf]

Supplementary Materials for

The Transcription Factor CsgD Contributes to Engineered *Escherichia coli* Resistance by Regulating Biofilm Formation and Stress Responses

Cheng-Hai Yan [1], Fang-Hui Chen [1], Yu-Lu Yang [1], Yu-Fan Zhan [1], Richard A. Herman [1], Lu-Chan Gong [1,2], Sheng Sheng [1,2] and Jun Wang [1,2,*]

1 Jiangsu Key Laboratory of Sericultural Biology and Biotechnology, School of Biotechnology, Jiangsu University of Science and Technology, Zhenjiang 212100, China; biojustych@163.com (C.-H.Y.); 212211802102@stu.just.edu.cn (F.-H.C.); 18770390337@163.com (Y.-L.Y.); 211111901120@stu.just.edu.cn (Y.-F.Z.); hermanansah44@yahoo.com (R.A.H.); lcgong@just.edu.cn (L.-C.G.); parasitoids@163.com (S.S.)

2 Key Laboratory of Silkworm and Mulberry Genetic Improvement, Ministry of Agriculture and Rural Affairs, Sericultural Research Institute, Chinese Academy of Agricultural Sciences, Zhenjiang 212100, China

* Correspondence: wangjun@just.edu.cn; Tel.: +86-511-85616571; Fax: +86-511-85620901

The biofilm of the parent strain and *csg*D deletion strain was stained, and the effect of crystal violet staining was observed and analyzed by optical microscope. As shown in Figure S1, compared with the parent strain, the biofilm formed by the *csg*D gene deletion strain has less crystal violet bound. And the density is also lower than that of the parent strains. Therefore, it is concluded that *csg*D gene regulates the growth of biofilm, and the deletion of *csg*D makes the content of biofilm decrease and the structure become looser.

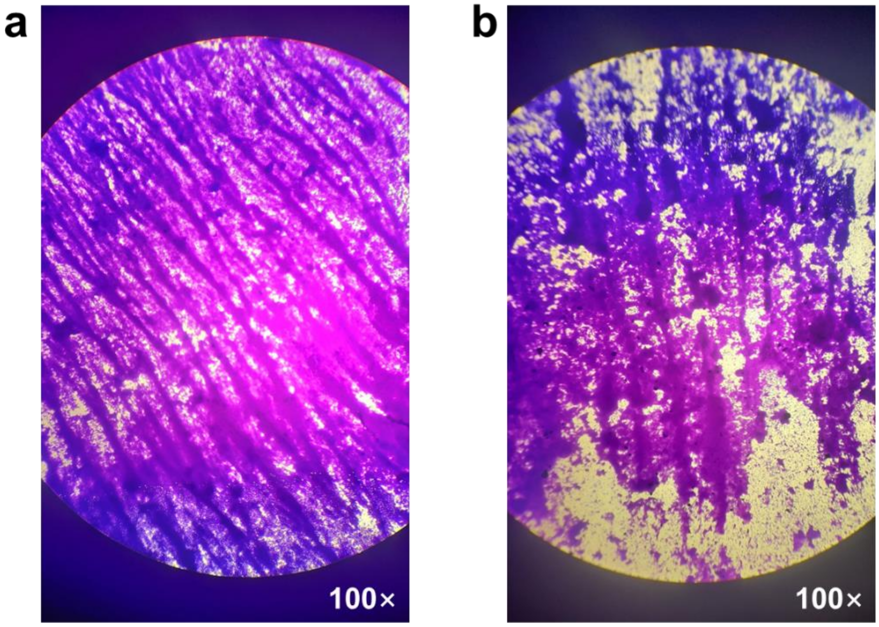

Figure S1. Light microscope observation (100×) of biofilms of the parent strains (a) and *csg*D gene deletion strains (b). The quantification of the biofilm was determined by crystal violet method.

The fluorescent dye SYBR Green I was combined with the molecules on the biofilm to form a fluorescent-labeled biofilm, and its green fluorescence was captured by fluorescent inverted microscope. As shown in Figure S2, the parent strain has strong fluorescence intensity and a large amount of biofilm adhesion. On the contrary, the amount of biofilm labeled by fluorescence in *csg*D-deficient strains decreased obviously, and the fluorescence intensity weakened, indicating that the adhesion of biofilm decreased and the thickness of biofilm became thinner. The above results further indicate that *csg*D gene has a significant positive regulatory effect on the growth of *E. coli* biofilm.

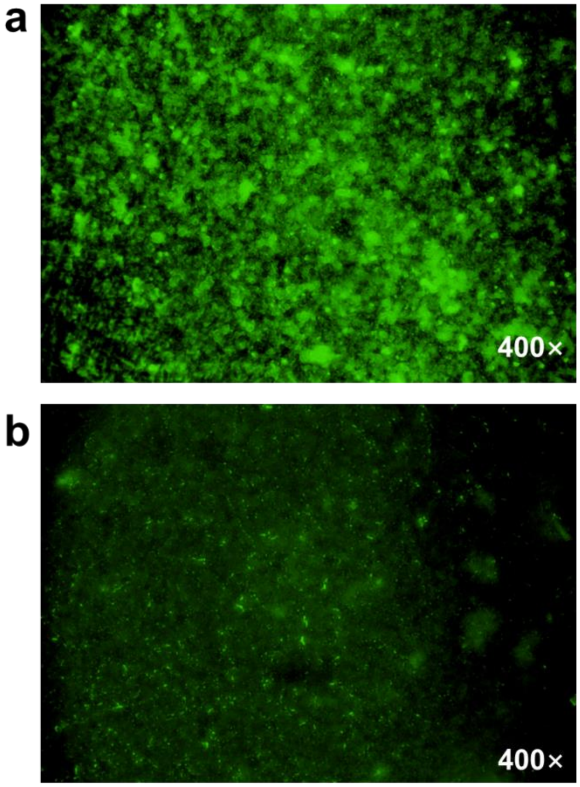

Figure S2. The ability of the parent strain *E. coli* BL21(DE3) (a) and *csg*D gene deletion strains (b) to form biofilms was tested by inverted fluorescence microscope (400×). The biofilms were stained with SYBR Green I dye, and observed by inverted fluorescence microscope.

Table S1. Strains and plasmids

Strains or plasmids	Characteristics	Source
Escherichia coli BL21(DE3)	Parent strain	Beijing TransGen Biotech Co., Ltd
Δ*csg*D	*csg*D deletion mutant strain	This study
pTargetF	Spectacomycin resistance, gRNA expression plasmid.	Wuhan Miaoling Biotechnology Co., Ltd.
PTargetF-sg-*csg*D	pTargetF contains sgRNA that targets *csg*D gene.	This study
pCas	Khanna resistance, expresses Cas9 recombinase, expresses λ-red recombinase, temperature-sensitive.	Wuhan Miaoling Biotechnology Co., Ltd.

Table S2. Primers for constructing *csg*D-deficient mutant strain

Primers	Primer sequence (5'-3')
*csg*D-F	TTATCGCCTGAGGTTATCGT
*csg*D-R	ATGTTTAATGAAGTCCATAG
pTarget-F	GTTTTAGAGCTAGAAATAGCAA
pTarget-R	CTTGTGATTATACCTAGGACTG
sgRNA	TAACTATCGTTATAACAGCA
sgRNA-*csg*D-F	GTCCTAGGTATAATCACAAGTAACTATCGTTATAACAGCAGTTTTAGAGCTAGAAATAG
sgRNA-*csg*D-R	CTATTTCTAGCTCTAAAACTGCTGTTATAACGATAGTTACTTGTGATTATACCTAGGAC
L. arm-F	TTTACCGGTATTAATATTCGACAGT
L. arm-R	CATGCCATGGAGGCGATAAAGCCATGAAAC
R. arm-F	CATGCCATGGAGGTTTAGTGATCAACAATAATGTA
R. arm-R	AAATATTTTTAATAACTCACCCGC
Cas9-F	TTATGGATTTAATTTAAACTTTTTATTTTAGGAGGCAAAA
Cas9-R	TCATGTCTAGATTAAGAAATAATCTTCATCTAAAATATAC

Table S3. Primers for differentially expressed genes *csg*D-deficient mutant strain

Primers	Primer sequence (5'-3')
lom-F	CATACCTCAGTGGCGTGGAG
lom-R	AATCCGTCAGTACGCCAGTC
*ftn*A-F	ACCGTTGAATCTCCGTTTGC
*ftn*A-R	GTCATTGCAGCATGAGCCAG
*nap*F-F	AGCGTCGGGCGAAAGATAAT
*nap*F-R	GGCCTGGGATTTGCAGTTCA
*ync*G-F	GTGGTTTTGACCACGAGGGA
*ync*G-R	GCCGCTGTCTCCGTCATAAT
*ent*D-F	GTTGACGAGATACCACGGCT
*ent*D-R	GGCTATAAATGTGTGCCCGC
*fep*A-F	TTGAATACGGGCTACGGTCG
*fep*A-R	GGAGTGGAATCAGCAACGGA
*ync*E-F	AGCTGATGATGCCACGAACA
*ync*E-R	TTACCGGTGTTCTGGATGGC
*ynf*M-F	CAACTTTCGTCTGCACTGGC
*ynf*M-R	GAGCATCAACCGATAGCCGA
*pga*A-F	CCTGGGGGCAATATTGGGTT
*pga*A-R	CATCGGATAAATCCGGGGCA
*mot*B-F	CGATGATTTCCCCTACGCCA
*mot*B-R	CGACACGTAACACTTTGCCG
*fim*A-F	ATCTAAAGCCGCTGTTGCCT
*fim*A-R	TCCAGGATCTGCACACCAAC
*fim*C-F	TTAGGTGCGACTCGCGTAAT
*fim*C-R	CACCATCGGCATTTTCCACC
*ira*P-F	ATGGCGCAAAATGACCAACA
*ira*P-R	CTTTACGTAATCGCGCAGCA
*omp*A-F	CCGGTTTCAGGGTTGCTTTG
*omp*A-R	GCGAAGCAGCTCCAGTAGTT
*osm*C-F	AAAGTGGATGCCGGTTTTGC
*osm*C-R	GGGCATCCTGCTTTTGCTTT
*suf*E-F	ACGGACGCACATCGAAATTG
*suf*E-R	GAATTACAGGGCGACAGCGA
*ela*B-F	CCTGCTTCGCCCGATAGTAA
*ela*B-R	CGATCCCGCCGATCAGAAATA

RNA-Sequencing Analysis

The *E. coli* BL21(DE3) and Δ*csg*D strains were cultured overnight, and the bacterial liquid was centrifuged at 8000 rpm for 10 min. The supernatant was removed, and the sample was frozen in liquid nitrogen. Three biological replicates were prepared for each treatment. The obtained samples were sent to Shanghai Biozeron Biotechnology Co., Ltd. for RNA-seq analysis. Total RNA was extracted from the tissue using TRIzol® Reagent according to the manufacturer's instructions (Invitrogen), and genomic DNA was removed using DNase I (TaKaRa). Then, RNA quality was determined using a 2100 Bioanalyzer (Agilent) and quantified using an ND-2000 (NanoDrop Technologies). RNA-seq strand-specific libraries were prepared following the TruSeq RNA sample preparation kit from Illumina (San Diego, CA) using 5 μg of total RNA. Briefly, rRNA was removed by a RiboZero rRNA removal kit (Epicenter) and fragmented using a fragmentation buffer. cDNA synthesis, end repair, A-base addition and ligation of the Illumina-indexed adaptors were performed according to Illumina's protocol. Libraries were then size selected for cDNA target fragments of 200-300 bp on 2% Low Range Ultra Agarose followed by PCR amplification using Phusion DNA polymerase (NEB) for 15 PCR cycles. After quantification by TBS380, paired-end libraries were sequenced by Illumina NovaSeq 6000 sequencing (150 bp*2, Shanghai BIOZERON Co., Ltd).

The raw paired-end reads were trimmed and quality controlled. Then, clean reads were separately aligned to the reference genome with *Escherichia coli* BL21(DE3) (CP053602.1). To identify DEGs (differentially expressed genes) between the two different samples, the expression level for each transcript was calculated using the fragments per kilobase of read per million mapped reads (RPKM) method. The DEGs between two samples were selected using the following criteria: the logarithm of fold change was greater than 2, and the false discovery rate (FDR) was less than 0.05
